# Correlation between the Results of Sequential Extraction and Effectiveness of Immobilization Treatment of Lead- and Cadmium-Contaminated Sediment

**DOI:** 10.1100/tsw.2010.13

**Published:** 2010-01-08

**Authors:** Milena B. Dalmacija, Miljana D. J. Prica, Bozo D. Dalmacija, Srdjan D. Roncevic, Ljiljana M. Rajic

**Affiliations:** ^1^ Faculty of Sciences and Mathematics, Department of Chemistry, Biochemistry and Environmental Protection, University of Novi Sad, Serbia; ^2^ Faculty of Technical Sciences, University of Novi Sad, Serbia

**Keywords:** sediment, sequential extraction procedure, remediation, stabilization/solidification, leaching test, leaching mechanism

## Abstract

The assessment of the quality of sediment from the Great Backi Canal (Serbia), based on the pseudo-total lead (Pb) and cadmium (Cd) content according to the corresponding Dutch standards and Canadian guidelines, showed its severe contamination with these two metals. A microwave-assisted BCR (Community Bureau of Reference of the Commission of the European Union) sequential extraction procedure was employed to assess their potential mobility and risk to the aquatic environment. Comparison of the results of sequential extraction and different criteria for sediment quality assessment has led to somewhat contradictory conclusions. Namely, while the results of sequential extraction showed that Cd comes under the high-risk category, Pb shows low risk to the environment, despite its high pseudo-total content. The contaminated sediment, irrespective of the different speciation of Pb and Cd, was subjected to the same immobilization, stabilization/solidification (S/S) treatments using kaolinite, montmorillonite, kaolinite-quicklime, montmorillonite-quicklime, fly ash, zeolite, or zeolite-fly ash combination. Semi-dynamic leaching tests were conducted for Pb- and Cd-contaminated sediment in order to assess the long-term leaching behavior of these metals. In order to simulate “worst case” leaching conditions, the semi-dynamic leaching test was modified using 0.014 M acetic acid (pH = 3.25) and humic acid solutions (20 mg TOC l^-1^) as leachants instead of deionized water. The effectiveness of S/S treatment was evaluated by determining diffusion coefficients (De) and leachability indices (LX). The standard toxicity characteristic leaching procedure (TCLP) was applied to evaluate the extraction potential of Pb and Cd. A diffusion-based model was used to elucidate the controlling leaching mechanisms. Generally, the test results indicated that all applied S/S treatments were effective in immobilizing Pb and Cd, and the treated sediments may be considered acceptable for “controlled utilization” based on LX values, irrespective of their different availability in the untreated samples. In the majority of samples, the controlling leaching mechanism appeared to be diffusion, which indicates that a slow leaching of Cd and Pb could be expected when the above S/S agents were applied. The TCLP results showed that all S/S samples were nonhazardous.

## INTRODUCTION

Anthropogenic, heavy metal contamination of various environments is a persistent problem in industrial societies. Therefore, sediments constitute reservoirs of potentially bioavailable metals that can lead to a bioaccumulation of toxic elements in the food chain, and induce perturbation of the ecosystem and adverse health effects[1,2]. It is now widely recognized that the toxicity and the mobility of these pollutants depend strongly on their specific chemical forms and on their binding state (precipitated with primary or secondary minerals, complexed by organic ligands, etc.). Indeed, changes in environmental conditions, such as acidification, changes in the redox potential conditions, or increases in organic ligand concentrations, can cause heavy metal mobilization from the solid to the liquid phase, and favor the contamination of surrounding waters. Hence, identification of the main binding sites and phase associations of heavy metals in soils and sediments helps in understanding geochemical processes in order to evaluate the remobilization potential and the risks induced[3].

Single and sequential extraction schemes have been designed for the determination of binding forms of trace metals in sediments[4]. These methods are based on the rational use of a series of more-or-less selective reagents chosen to solubilize successively the different mineralogical fractions thought to be responsible for retaining the larger part of the trace elements. They are intended to simulate the various possible natural and anthropogenic modifications of environmental conditions[5].

Many of the sequential extraction methods are, in fact, variants on the Tessier procedure[6], in which the exchangeable metals and those nominally associated with carbonate, Fe-Mn oxides, organic material, and silicate residues are extracted with different reagents[5]. Based on the Tessier procedure, the Community Bureau of Reference of the Commission of the European Union produced the definition of an extraction protocol (the BCR protocol) and a purely operational definition of sequential fractionation. This method partitions the heavy metals into four fractions: exchangeable and carbonate bound, iron and manganese oxides bound, organic matter bound, and residual metal. However, extraction schemes have been widely criticized and the great variety of protocols that have been developed reflects the complexity of the problems: lack of uniformity in the procedures, lack of selectivity of the reagents used, lack of quality control, results highly dependent on the procedure used, etc. The choice of procedure must be related to a definite objective, taking into account the nature of the sample: sediment, soil, sludge, or industrially polluted soil. One of the main limitations of sequential extraction procedures is that they are extremely time consuming, so that they are less used for routine analysis. This could be improved by using microwave (MW) irradiation or its combination with ultrasonic shaking, to accelerate different chemical processes, including multistep sequential extraction. MW heating has also been used successfully to accelerate the sample preparation process[7].

Despite all the criticisms, the sequential extraction schemes remain widely used and are considered an essential tool in establishing element fractionation in soils and sediments[5]. Finally, in the not-too-remote future, the combination of the application of well-designed sequential extraction schemes and speciation studies of the solutions obtained will give a better view of the potential for transfer of trace elements in the environment and of the risks involved.

The distribution of metals in different phases obtained by sequential extraction procedures offers an indication of their availability, which, in turn, allows the assessment of the risk of their presence in the aquatic environment. The Risk Assessment Code (RAC) gives an idea of the possible risk by applying a scale to the percentage of metals presented in exchangeable and carbonate fractions, i.e., labile phases. According to the RAC, if this fraction is <1%, there is no risk for the aquatic system, 1–10% exhibits low risk, 11–30% medium risk, 31–50% high risk, and >75% very high risk[8].

Various technologies have been developed to transform hazardous wastes to nontoxic, or reduce the potential release of toxic species into the environment. The solidification/stabilization (S/S) treatment was used to treat nuclear wastes in the 1950s and then was widely applied to hazardous wastes in the early 1970s[9]. S/S has been identified by the U.S. Environmental Protection Agency (EPA) as the Best Demonstrated Available Technology for 57 regulated hazardous wastes[10] and it is one of the most commonly applied technologies at Superfund sites in the U.S., used at 24% of the sites between 1982 and 2002.

The S/S techniques have been widely applied to treat soils with heavy metal contamination. The hazardous waste potential can be minimized by converting the contaminants into forms that are less soluble, less mobile, or less toxic, or by encapsulating the waste within a monolithic solid of high structural integrity[9]. Various combinations of stabilizing agents have been used to treat soils contaminated with lead (Pb). Jing et al.[11] studied Pb leachability from cement, lime, and fly ash S/S soil samples. Dermatas and Meng[12] used quicklime and fly ash to evaluate the degree of Pb immobilization using the toxicity characteristic leaching procedure (TCLP). Li et al.[13] studied Pb immobilization using Portland cement and pulverized fly ash. Long and Zhang[14] used cement in combination with various additives, such as lime, fly ash, clay, apatite, and silicate for treating Pb-contaminated soils.

At the moment, there are not enough data about S/S treatments of contaminated sediments, especially those contaminated with cadmium (Cd), and the conclusions obtained for soils cannot be valid in full for sediments.

Owing to its pozzolanic nature, fly ash can be used in a variety of construction applications. Namely, combustion of subbituminous coal produces a fly ash (fly ash class C) rich in calcium, with self-cementing characteristics, and the pozzolanic reactions lead to calcium aluminum and calcium silicate hydrate (CAH and CSH) cementations product formation. It has been reported that 26% of the total quantity of fly ash produced annually in the U.S. is used in construction materials[12,15]. In Serbia, there is still no use of fly ash at all.

In the past 2 decades, research has been focused on using low-cost effective sorbents for heavy metal adsorption. Natural materials like clay and zeolite have been investigated as potential adsorbents for this purpose. The capability of zeolites to exchange cations is one of their most useful properties, and it determines their ability to remove heavy metals from industrial wastewaters[16,17].

The degree of effectiveness of the S/S products is defined basically by two parameters: strength and leach resistance. Leaching is known to be a complex phenomenon because many factors may influence the release of specific constituents from a waste over a period of time. These factors include major element chemistry, pH, redox potential, complexation, liquid-to-solid ratio, contact time, etc.[18]. Moreover, since very little is known about the chemical species present in waste forms and their behavior with respect to time, the long-term performance of S/S waste forms has been difficult to predict. In order to predict the long-term leaching behavior, a diffusion model is frequently used to evaluate the leaching kinetics. The mechanisms governing heavy metal leachability in quicklime-based solids can be effectively examined using the American Nuclear Society's (ANS) semi-dynamic leaching test[19]. The ANS 16.1 provides substantially more information regarding the “real time” rate at which heavy metals are released from the solidified product as compared to other leaching tests[12]. The leaching results extend over a 90-day period instead of a single result at the end of the test. The most often used leaching test, recommended by the EPA, but which provides only one result for defining the waste toxicity, is the TCLP test[20]. The TCLP was specifically designed to mimic acidic conditions in a sanitary landfill and identify wastes that have the potential to contaminate groundwater.

In view of the above, the objectives of this study were to (1) define Pb and Cd speciation in sediment and evaluate their environmental risk, (2) evaluate the effectiveness of S/S treatments (with the addition of kaolinite, montmorillonite, quicklime, fly ash, and zeolite), (3) evaluate the effectiveness of S/S treatments in conditions that mimic the landfill environment, (4) determine the controlling leaching mechanisms of Pb and Cd from untreated and treated sediment samples using deionized water as a leaching solution, and (5) correlate the results of sequential extraction procedure and S/S treatments.

## Evaluation of Leaching Behavior: Diffusion Model

We evaluated the long-term leachability of heavy metals from the S/S-treated sediments using the ANS 16.1 method[19]. By applying this test, we get the cumulative fraction of metals leached vs. time. A mathematical diffusion model based on Fick's second law is used to evaluate the leaching rate as a function of time. The ANS has standardized the Fick's law–based mathematical diffusion model as follows[21]:

$$\text{De} = \pi \left[ \frac{\frac{a_n}{A_0}}{(\Delta t)_n} \right]^2 \left[ \frac{V}{S} \right]^2 T_n \qquad (1)$$

where $a_n$ is the contaminant loss (mg) during the particular leaching period with subscript $n$, $A_0$ is the initial amount of contaminant present in the specimen (mg), $V$ is the specimen volume (cm$^3$), $S$ is the surface area of specimen (cm$^2$), $\Delta tn$ is the duration of the leaching period in seconds, $Tn$ is the time that elapsed to the middle of the leaching period $n$ (sec), and De is the effective diffusion coefficient (cm$^2$ s$^{-1}$).

The De value in Eq. 1 is termed "effective" because the diffusion occurs in the liquid filling the interstitial space of a porous body. Therefore, the actual liquid path is longer than the one assumed by the model. The exact solution of the diffusion equation depends on the initial and boundary conditions. Due to the slow diffusion rate of contaminants, it can be assumed that kaolinite-, montmorillonite-, quicklime-, zeolite-, and fly ash–based waste forms are semi-infinite media, much like the cement-based waste forms examined in previous studies[15]. This implies that the release of the contaminant from the waste form is negligible when compared to the contaminant's total mass. As a result of this implication, diffusion is expected to be the controlling leaching mechanism in sediments treated with these S/S agents.

The leachability index (LX) is a parameter directly derived from the ANS 16.1 test results. It is calculated using the diffusion coefficient obtained from Eq. 1. It is the average of the negative logarithm of the effective diffusivity terms (expressed in cm$^2$ s$^{-1}$). Therefore, the LX is defined as follows:

$$\text{LX} = \frac{1}{m} \sum_{n=1}^{m} \left[ -\log(D_e) \right]_n \qquad (2)$$

where $n$ is the number of the particular leaching period and $m$ is the total number of individual leaching periods. The relative mobility of different contaminants can be evaluated by this index, which varies from 5 (De = $10^{-5}$ cm$^2$s$^{-1}$, very mobile) to 15 (De = $10^{-15}$ cm$^2$s$^{-1}$, immobile)[22].

LX is currently used by Environment Canada[23] as a performance criterion for the utilization and disposal of treated waste. Treatment is considered effective if the LX value of treated waste is higher than 9 and S/S wastes are acceptable for a specific utilization such as quarry rehabilitation, lagoon closure, roadbase material, etc. If the S/S wastes have an LX value higher than 8, they can be disposed of in segregated or sanitary landfills. If the S/S wastes have an LX value lower than 8, they are not considered appropriate for disposal.

## Determination of the Controlling Leaching Mechanism

The type of leaching mechanism that controls the release of metals can be determined based on the values of the slope of the logarithm of cumulative fraction release, log($Bt$), vs. the logarithm of time, log($t$), line[18]. If diffusion is the dominant mechanism, then theory suggests the following relationship:

$$\log(B_t) = \frac{1}{2}\log(t) + \log\left[U_{\max} d \sqrt{\left(\frac{D_e}{\pi}\right)}\right] \quad (3)$$

where De is the effective diffusion coefficient in $m^2\ s^{-1}$ for component *x*, *Bt* is the cumulative maximum release of the component in mg $m^{-2}$, *t* is the contact time in seconds, $U_{max}$ is the maximum leachable quantity in mg $kg^{-1}$, and *d* is the bulk density of the product in kg $m^{-3}$.

When the slope is close to 1, according to de Groot and van der Sloot[18], the process is defined as dissolution. In that case, the dissolution of the material from the surface proceeds faster than the diffusion through the pore space of the soil matrix. If the slope is 0.5, the release of heavy metals will be slow and diffusion will be the controlling mechanism. Typically, the long-term leaching characteristics of S/S-treated wastes are controlled by diffusion. However, there are cases when the other processes, dissolution and wash-off, may also occur[15].

### TCLP Leaching Procedure

At the present time, TCLP is used by the EPA to evaluate whether a particular S/S process is effective in treating a given waste, in the sense of having toxicity characteristics[20].

## MATERIALS AND METHODS

### Sediment Samples, Reagents, and Materials

Sediment samples were collected from the Great Backi Canal (Vojvodina, Serbia). The top sediment sample was taken with an Eijkelkamp core sampler in the middle of the canal (water depth ranging from 2.0 to 5.5 m). The results of sediment metal pseudo-total concentrations presented in the study are discussed in reference to Dutch Regulation Standards[24] and Canadian Sediment Quality Guidelines[25], since Serbia has neither an established system of continual monitoring of sediment quality nor regulations concerning the quality standards. Pseudo-total trace metal contents were assessed on sample triplicate after aqua regia digestion[26] and mean values were used. The relative standard deviations (% RSD) obtained (n = 3) were below 10%. According to the Dutch system, the class limits are defined for "standard sediment", with 10% organic matter and 25% clay. In assessing sediment quality, the values for a standard sediment are converted to the values applying to the actual sediment concerned on the basis of the measured organic material (measured by percentage weight loss by volatilization, on the total dry weight of the sediment) and clay content (the percentage by weight of total dry material comprising mineral particle matter with a diameter smaller than 2 µm). Metal concentrations of the sediment were first corrected to standard sediment based on correction formulas and then classified according to the Dutch national evaluation scheme[24]. Finally, the overall sediment quality was established using the "worst class" sediment parameters.

The MW-assisted sequential extraction (MSE) procedure was performed as described by Jamali et al.[27], using identical operating conditions applied in each individual BCR fraction and validated by the CRM BCR 701. Mean values were used and the RSD (n = 3) were below 5%. The extractions were performed at ambient temperature and the optimization of the MW power and extraction time was carried out by carefully controlling the temperature of the extracting solutions, which did not exceed 50°C, and the solutions were never brought to boiling. Blanks (containing reagent, but no samples) were also taken through each complete procedure. Mileston, Stare E microwave (MW) was used for MW extraction and digestion.

The extracting solutions were prepared from analytical-grade reagents. Standard solutions of Cd and Pb were prepared by diluting the 1000 ppm certified standard solutions, Fluka Kamica (Buchs,

Switzerland), of the corresponding metal ions. The hydroxylammonium chloride solution was prepared prior to use. The certified reference material BCR 701 was purchased from the EU Bureau of References. All glassware and plastic materials used were previously treated for 24 h in 2 *M* nitric acid and rinsed with deionized water. Extraction was carried out using 100-ml acid-washed polyethylene centrifuge tubes, while 100-ml polyethylene vessels were used for storing the extracts.

The pseudo-total metal contents obtained by aqua regia digestion of the BCR 701 and sediment sample were compared with the sum of the extracted metals forms (the three steps plus residual). No significant difference was observed between the total metal extracted following the aqua regia protocol and the sum of extracted metals following MSE procedures. The concentrations of Pb and Cd extracted from the reference material differed by 2.5% for Pb and 1.5% for Cd, and the untreated sediment sample differed by 7% for Pb and by 5% for Cd, which is in accordance with the literature data[28]. The good agreement observed with the results obtained as the sum of the four steps and the pseudo-total content shows that the laboratory working conditions were under control. No significant difference in extracted concentrations of three BCR 701 and sediment sample probes (RSD <5%) was observed.

Pseudo-total sediment metal content and metal content in sequential extraction steps were determined by AAS (Perkin Elmer AAnalyst™ 700) or ICP-MS (Perkin Elmer Sciex Elan 5000) according to the standard procedures[29,30]. All the results are expressed with respect to sediment dry matter.

## Sample Preparation for S/S Treatments: ANS 16.1 Test

Sediment samples of an average initial moisture content of 65% were dried at 105°C to the constant mass and then mixed with the following immobilization agents: fly ash class C, natural zeolite, kaolinite, montmorillonite, and quicklime. Specimens were designated by the capital letters, i.e., K: kaolinite, M: montmorillonite, Z: zeolite, F: fly ash, L: quicklime, followed by a number indicating the percent weight of the given attribute. The S/S agent content was expressed as percentage of the total solids weight. Additionally, the amount of S/S agents present was varied in order to evaluate its relative contribution to Pb and Cd immobilization. During the leaching test, 21 types of specimens were tested: K10, K20, K30, M10, M20, M30, K10L10, K20L10, K30L10, M10L10, M20L10, M30L0, Z10, Z20, Z30, F10, F20, F30, Z5F10, Z10F10, and Z5F20.

Class C coal fly ash was provided from the Kolubara thermal power plant and its composition was (% wt.): $SiO_2$ (39.4), $Al_2O_3$ (20.1), $Fe_2O_3$ (4.95), MgO (4.01), CaO (23.2), $K_2O$ (0.64), $Na_2O$ (2.12), $SO_3$ (1.88). The zeolite (clinoptilolite) composition was as follows (% wt.): $SiO_2$ (66.9), $Al_2O_3$ (13.5), $Fe_2O_3$ (0.98), MgO (0.69), CaO (3.85), $K_2O$ (0.54), $Na_2O$ (0.37), $SO_3$ (1.18) and ignition loss (11.4). Two types of clay minerals, kaolinite ($SiO_2$ [45.9], $Al_2O_3$ [37.2], $Fe_2O_3$ [3.34], MgO [1.40], CaO [0.25], $K_2O$ [0.14], $Na_2O$ [0.10], ignition loss [13.3]) and montmorillonite ($SiO_2$ [58.9], $Al_2O_3$ [22.7], $Fe_2O_3$ [4.83], MgO [1.40], CaO [1.85], $K_2O$ [0.24], $Na_2O$ [0.12], ignition loss [10.6]), were selected in order to represent the two extremes of physicochemical clay behavior based on their surface area and cation exchange capacity (CEC). The CEC (meq/100 g) was 5.4 for kaolinite and 90.6 for montmorillonite, while the respective surface areas ($m^2$ $g^{-1}$) were 66 and 720. Thus, the effect of relatively nonreactive clay (kaolinite) on Cd and Pb immobilization was compared with that of the highly reactive clay (montmorillonite).

Samples were prepared in the form of monolithic cubes [(3 ± 0.1) × (3 ± 0.1) × (3 ± 0.1) cm] by compaction at an optimum water content, defined as the water content at which the maximum dry density is achieved for a given compactive effort. The compaction was performed according to ASTM D1557-00, providing a compactive effort of 2700 kN m $m^{-3}$[19]. Samples were cured at 20°C in sealed sample bags for 28 days and then subjected to the ANS 16.1 test. Prior to the ANS 16.1 test, loose particles present on the solid's surface were rinsed out by immersing the solid in distilled water for 30 sec. In order to suspend each specimen near the centroid of the solution, a nylon mesh harness was used to support the specimen in a polyethylene container.

In this study, the ANS 16.1 method was modified by including 0.014 *M* acetic acid (AA) pH 3.25 (similar to the TCLP procedure) and humic acid (HA) solutions (20 mg TOC $l^{-1}$) as leachants. The objective was to mimic the worst possible conditions of the S/S waste disposed in the landfill environment.

According to the ANS 16.1 test[19], the liquid/solid ratio was 10:1 (l $kg^{-1}$). The leachate was collected and replaced at defined time intervals (2, 7, 24, 48, 72, 96, 120, 456, 1128, and 2160 h). The leachates were filtered through a 0.45-µm pore size membrane filter and then analyzed for Cd and Pb on AA700 Perkin Elmer atomic absorption spectrometer and an Elan 5000 ICP-MS instrument. All materials in contact with the leachant were precleaned with $HNO_3$ and subsequently rinsed with deionized water.

Only for the mixture K10, the ANS 16.1 semi-dynamic test was performed in triplicate and mean values are presented, the RSD (n = 3) being below 10%.

At the end of the 28-day curing period, the standard TCLP leaching method (EPA, 1996) was applied on every sample in triplicate and the RSD were below 5%.

Determination of the controlling leaching mechanism from the specimens was done according to the diffusion model used by de Groot and van der Sloot[18], represented by Eq. 3. De is the effective diffusion coefficient ($m^2\,s^{-1}$) for Pb and Cd obtained from the ANS 16.1 test and described by Eq. 1, *Bt* is the calculated cumulative maximum release of the Pb and Cd (mg $m^{-2}$), *t* is the contact time in seconds (2, 7, 24, 48, 72, 96, 120, 456, 1128, and 2160 h converted in seconds), $U_{max}$ is the maximum leachable quantity (mg $kg^{-1}$), and *d* is the bulk density of the specimen (kg $m^{-3}$).

Logarithm of the cumulative release, log(*Bt*), is plotted vs. the logarithm of time, log(*t*), and linear regression was done. Depending on the slope of the line at different time periods, the process of transport is differentiated as surface wash-off, diffusion, or dissolution.

## RESULTS AND DISCUSSION

### Pseudo-Total Metal Concentrations and Sequential Extraction of Untreated Sample

The assessment of the sediment quality based on the pseudo-total Pb and Cd content according to the corresponding Dutch standards[24] and Canadian guidelines[25] showed severe contamination. However, it is now widely accepted that the role of aquatic sediments as a sink or a source of pollutants cannot be fully assessed by measuring pseudo-total metal concentrations[3], as they do not give an accurate estimation of the likely environmental impact, as shown in our work. The mobility of trace metals, as well as their bioavailability and related ecotoxicity to plants, critically depends upon the chemical form in which a metal is present in the sediment[5]. According to sequential extraction, the first fraction is the most dangerous for the environment and a significant proportion of Cd and negligible proportion of Pb is present in this fraction. According to the risk assessment connected with the metal available in the first phase of sequential procedure, Cd comes under the high-risk category and Pb shows low risk to the environment, which is not in accordance with the assessment of the sediment quality based on the Dutch standards and Canadian guidelines.

The pseudo-total contents of Pb and Cd in the untreated sediment sample were 1219 and 25.52 mg $kg^{-1}$, respectively. The results of sediment metal pseudo-total concentrations presented in the study are discussed in reference to the Dutch standards[24] and the Canadian guidelines[25], since Serbia has neither an established system of continual monitoring of sediment quality nor regulations concerning the quality standards.

According to the Dutch regulation standards[24], pseudo-total Pb and Cd content is above intervention value and such sediment is considered severely polluted with Pb and Cd (class 4) and needs dredging, disposal in special reservoirs, and, if possible, sediment cleanup measures.

Compared with the Canadian guidelines[25] for aquatic life protection, contents of Cd and Pb were above the probable effect level (PEL). Sediment concentrations above PEL values are expected to be frequently associated with adverse biological effects. Although PEL is considered to be applicable to a variety of sediment types, it cannot define uniform values of sediment pollution, as the bioavailability (and hence toxicity) of contaminants can differ[25].

The distribution of Pb and Cd in different phases extracted by the MSE procedure is shown in Fig. 1. The first fraction is the most dangerous for the environment and a significant proportion of Cd (37.70%) is present in the exchangable forms and bound to carbonates. Metals in exchangeable fraction can be exchanged and are in equilibrium with the ionic content in water. The carbonates fraction mainly refers to the metals that are precipitated or coprecipitated with carbonate. This fraction is sensitive to pH variations. The fractionation profile of Cd shows that its large portion is also bound to reducible phases, Fe and Mn oxides (60.87%). The Fe-Mn oxides fraction includes the soluble metal oxides/hydroxides under slightly acidic pH as well as the metal associated with reducible amorphous Fe-Mn oxyhydroxides. This fraction can be dissolved with oxidation-reduction potential change. The fractionation profile of Pb suggests that the major portion of Pb is associated with residual fraction (90.60%), while a negligible portion is present in the environmentally most dangerous phase (1.34%). The residual fraction represents the stable metal forms associated with anthropogenic or geogenic components, the influence of which on the ecological system is much smaller than the others under the majority of conditions[5]. Other studies also reported similar Cd and Pb fractionation[27,31,32,33].

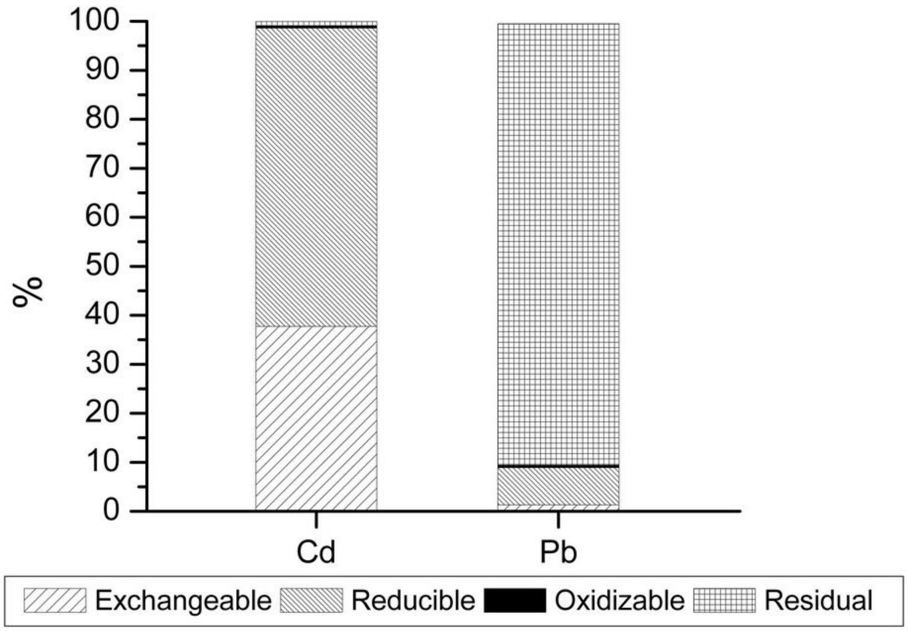

**FIGURE 1**. Distribution of Cd and Pb in sequentially extracted fractions of untreated sediment sample.

It is evident from the results of the fractionation study that the metals in the sediments are bound to different fractions with different strengths. The risk connected with the type of association, or the type of bounding of metals in sediment, is described by the RAC. This criterion indicates that a sediment that can release in exchangeable and carbonate fractions <1% of the total metal will be considered safe for the environment. On the contrary, a sediment releasing in the same fraction >50% of the total metal has to be considered highly dangerous and can easily enter the food chain[8]. According to the RAC, 37.70% of Cd in carbonate phases comes under the high-risk category and can easily enter the food chain. Because of its toxicity and availability, Cd can cause serious problems to the ecosystem. On the other hand, according to this code, Pb shows low risk to the environment with 1.34% in carbonate fraction.

The results of sequential extraction are not in full agreement with the results of pseudo-total metal concentration in the sediment, which only confirms the opinion that the total metal concentration is not sufficient to define the real danger to the environment. Based on the pseudo-total metals concentration, the sediment is of Class 4 (Dutch standards) and above Canadian guidelines in respect of both metals, and is considered as highly contaminated. However, judging from the results of sequential extraction, the Pb is present in the environmentally least dangerous phase, and hence cannot be considered as dangerous to the environment. As far as Cd is concerned, based on both Canadian and Dutch recommendations, it can be concluded that its concentration in the sediment is dangerous to the environment. These results obviously have to be taken into account in the assessment of the sediment quality, remediation procedures, and sediment disposal in general.

## Sequential Extraction of Treated Samples Prior to the ANS 16.1 Leaching Test

The ranking of metals in first phase of the MSE procedure (Fig. 2) according to their percentage contribution was in the following range for Pb: 0.13% (Z10F20) to 4.1% (Z30), while for Cd, it was from 20.1% (Z10F20) to 65.3% (K10L10). Based on the percentage of metals extracted in the first phase of sequential extraction, Pb was most soluble in the following forms: M10L10, Z30, F10, F20, F30, and K5F10, even though in very low percentages, while Cd solubility was notable in almost all samples. Pb content in residual fraction was in the range 54.4% (M10) to 92.9% (M30L10) and for Cd 0.3% (Z20) to 9.7% (M20).

Comparison of the results (Figs. 1 and 2) of sequential extraction of treated and untreated samples shows that the Pb content in the residual phase decreased by 2% (M20L10) to 30% (M10), but still remained high compared to the Cd content in this inert fraction: 0.37% (Z20) to 9.77% (M20). The Cd content in reducible phase decreased by 38% (K30L10) to 93% (K5F10). In the exchangeable fraction, it increased its content in some fractions (K10L10, F30, K5F10), even by 40%. Similar results were obtained for the Pb-contaminated soil treated with cement, quicklime, and fly ash[34].

A potential method to determine if the heavy metals can be removed by remediation techniques or predict removal efficiency is to determine speciation with selective extraction techniques[35]. It is believed that the exchangeable, carbonate, and reducible oxide fractions may be amenable to soil washing techniques[36]. Removal of the organically and residually bound fraction may be justified if, as in our case, one metal is easily available, i.e., present in the exchangeable and reducible fractions (Cd) and the other is potentially low available (Pb). Gombert[37] used sequential extraction to determine if cesium, cobalt, and chromium could be removed by soil washing. Since <20% was extracted after dissolving 20% of the soil mass, soil washing was abandoned as an option. Mulligan et al.[38] demonstrated that the sequential extraction procedure could be used prior to soil washing, to design and monitor the remediation process.

Although Pb and Cd speciations in sediment were different, we applied the same remediation treatment because there are not enough data about the behavior of metals differently distributed in their mixture in a sediment during their S/S treatment, and about the treatment efficiency in general. The main objective of every remediation procedure dealing with several contaminants is to carry out the treatment with the same agents, and thus achieve economic and environmental benefits.

## Cumulative Release of Cd and Pb Before and After S/S Treatment using Deionized Water as Leachant

The cumulative values of Pb and Cd leachability from the specimens treated with kaolinite, montmorillonite, zeolites, fly ash, and kaolinite-quicklime, montmorillonite-quicklime, and zeolites-fly ash combinations are presented in Table 1.

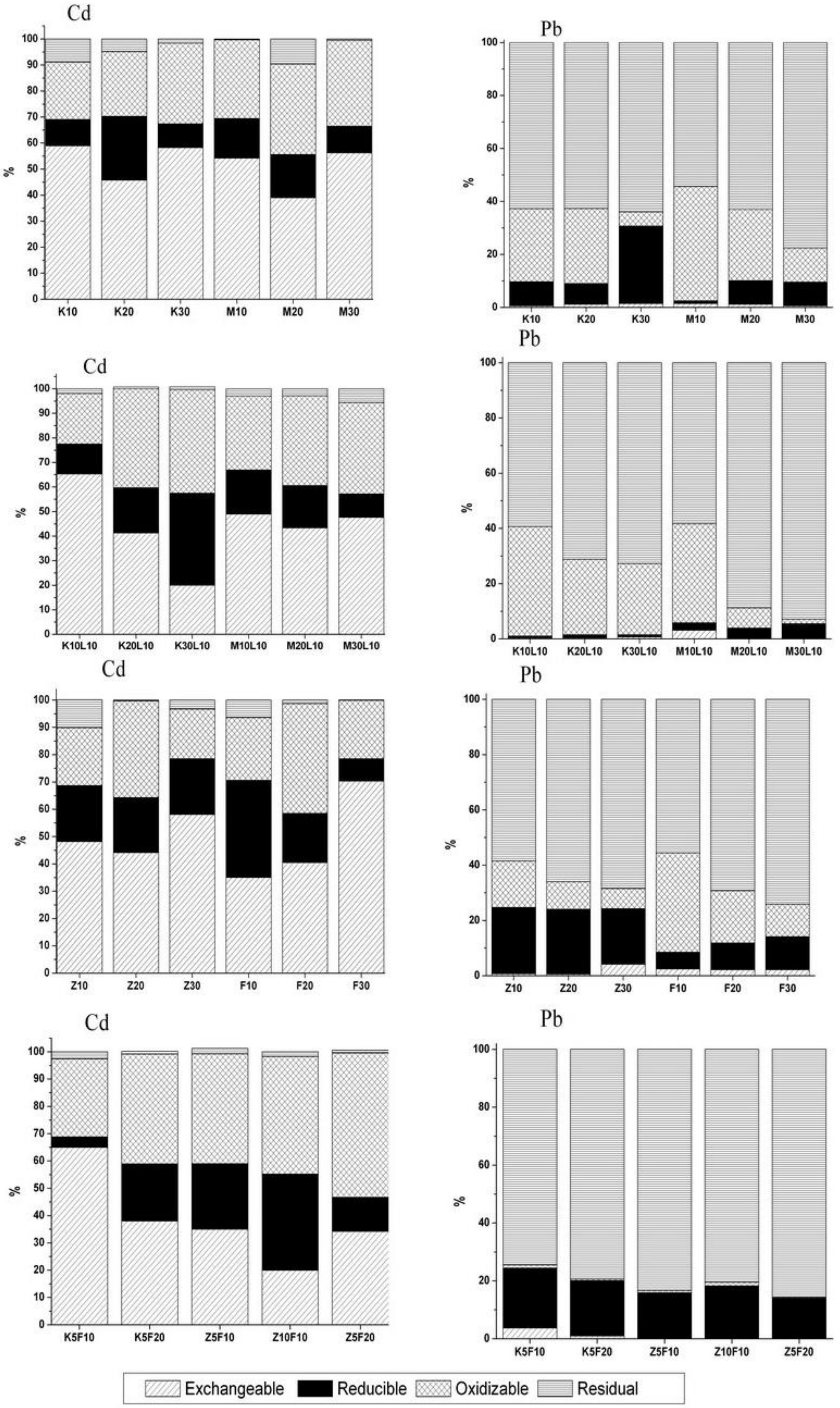

**FIGURE 2.** Distribution of Cd and Pb in sequentially extracted fractions of treated sediment samples before the ANS 16.1 leaching test.

**TABLE 1**
**Total Cumulative Fraction (%) of Cd and Pb Leached in ANS 16.1 using Deionized Water (DI Water), AA pH 3.25, and HA 20 mg TOC $l^{-1}$ as Leachants**

| | Cd (%) | | | Pb (%) | | |
|---|---|---|---|---|---|---|
| | DI | AA pH 3.25 | HA 20 mg TOC $l^{-1}$ | DI | AA pH 3.25 | HA 20 mg TOC $l^{-1}$ |
| Untreated SS* | 17.6 | 22.3 | 23.1 | 1.10 | 15.2 | 10.8 |
| K10 | 1.20 | 4.03 | 4.10 | 0.27 | 1.07 | 0.07 |
| K20 | 1.17 | 4.17 | 4.47 | 0.25 | 0.46 | 0.07 |
| K30 | 1.07 | 4.48 | 4.88 | 0.14 | 0.16 | 0.05 |
| M10 | 1.03 | 3.88 | 4.04 | 0.14 | 0.18 | 0.06 |
| M20 | 0.76 | 4.21 | 4.41 | 0.08 | 0.15 | 0.05 |
| M30 | 0.19 | 4.55 | 4.60 | 0.07 | 0.11 | 0.04 |
| K10L10 | 1.85 | 3.52 | 4.12 | 0.23 | 0.30 | 0.07 |
| K20L10 | 1.73 | 3.25 | 3.69 | 0.21 | 0.25 | 0.09 |
| K30L10 | 3.06 | 6.41 | 6.50 | 0.13 | 0.22 | 0.09 |
| M10L10 | 2.10 | 3.84 | 3.87 | 0.15 | 0.19 | 0.04 |
| M20L10 | 2.22 | 3.45 | 4.22 | 0.07 | 0.12 | 0.02 |
| M30L10 | 2.18 | 3.62 | 4.16 | 0.03 | 0.12 | 0.01 |
| Z10 | 1.39 | 4.08 | 4.28 | 0.09 | 0.15 | 0.07 |
| Z20 | 1.34 | 4.75 | 4.84 | 0.09 | 0.17 | 0.06 |
| Z30 | 1.17 | 3.47 | 3.65 | 0.09 | 0.12 | 0.08 |
| F10 | 1.69 | 4.09 | 4.08 | 0.08 | 0.18 | 0.08 |
| F20 | 1.77 | 4.29 | 4.41 | 0.03 | 0.18 | 0.08 |
| F30 | 2.02 | 4.69 | 4.99 | 0.17 | 0.20 | 0.09 |
| Z5F10 | 2.45 | 4.58 | 4.15 | 0.07 | 0.24 | 0.05 |
| Z10F10 | 2.34 | 4.97 | 4.28 | 0.03 | 0.19 | 0.07 |
| Z5F20 | 2.60 | 4.63 | 4.42 | 0.03 | 0.17 | 0.05 |

* Untreated SS, untreated sediment sample.

The clays employed exhibited good sorption of Pb and Cd, reducing significantly their leachability compared to untreated sample (up to 99%). There are not enough data about sorption of sediment metals on clays, but some studies mentioned their good sorption properties with respect to soil metals[15,39,40]. In the case of treated samples (Table 1), the Pb and Cd leachability is influenced by the clay type. Montmorillonite-treated samples showed a pronounced reduction in Pb and Cd leachability compared to those treated with kaolinite. This is most probably due to the larger surface area and the greater CEC of montmorillonite. Judging from the percentages of metals leached, the mixture M30 appeared to be best for stabilization of Pb (0.07) and Cd (0.19). This is in a good correlation with the literature data[15,40] for natural and artificially prepared soils in case of Pb leaching. Generally, with the small differences between mixtures, there is still a very low cumulative percentage of metals leached. If compared with the kaolinite-quicklime and montmorillonite-quicklime treatments, it is clear that a very low cumulative Pb and Cd fraction (<3%) was leached out within this time period when the deionized water was used as a leachant. Quicklime addition to the mixtures with montmorillonite (M20L10, M30L10) showed improvement if compared with the other samples (M10, M20, M30, M10L10) for Pb and a good correlation with the literature data[41].

The zeolite-treated samples (Table 1) showed a pronounced reduction (by 95 to 99%) in Pb and Cd leachability compared to untreated samples. An increased amount of zeolites led to decreased amounts of Cd and Pb leached from the samples (cumulative percentages leached ranged from 1.39 to 0.09%). This might be due to the greater availability of the exchangeable adsorption sites at the available larger sorbent surface area, noticed also in some other studies, but only for Cd[42]. As expected, the removal efficiency of cationic species is very good, which can be ascribed to cation exchange and electrostatic interactions with the negative surface charge of the natural zeolite. It is noted that the exchangeable ions in natural zeolites predominantly are $Na^+$, $Ca^{2+}$, and $K^+$, and thus can be favorably replaced by charged Cd and Pb ions via cation exchange[43]. Zeolites showed better sorption capacity for Pb, which agrees with the literature data[44,45,46]. The combination of zeolites with fly ash (Z5F10, Z10F10, Z5F20) yielded no improvement of Cd stabilization compared with the treatments involving only zeolites or fly ash, as the percentages of leached metals were in the range from 2.45 to 2.60. However, the decrease in the Pb leachability was significant in the combined treatment with zeolites and fly ash (by about 99%). The increase of the proportion of fly ash yielded a decrease in the percentage of leached Pb.

Since fly ash is by itself considered a waste, its use to treat contaminated media would be, at the same time, a cost-effective method of its disposal and mitigating possible negative environmental effects, originating in either the fly ash or the solid waste, through proper engineering control. Upon sole addition of fly ash, Pb and Cd leachability was decreased (cumulative percentages leached were <2% for Cd and <1% for Pb) (Table 1). Fly ash class C showed a pronounced reduction in Pb and Cd leachability compared to untreated samples (reduction by 90 to 99%). This may be due to the formation of pozzolanic reaction products, such as CSH and CAH, capable of stabilizing metal cations[47]. Therefore, it is plausible to think that these pozzolanic reaction products will contribute to Pb and Cd immobilization by sorption and/or chemical inclusion. An increased amount of fly ash led to decreased amounts of Pb leached from the samples. Also, if compared with the literature data, our cumulative percentages of leached Pb are smaller than in the case when fly ash was used as an S/S agent in the treatment of contaminated soil[12]. Fly ash showed better sorption capacity for Pb and with the increasing fly ash content (until F20), the leachibility decreased, which is in agreement with the literature data[15,48] for natural and artificially prepared soils in case of Pb leaching. This may be a result of the formation of insoluble Pb silicate precipitates or pozzolanic reaction products, or both. Moon and Dermatas[15] reported that Pb immobilization in quicklime-fly ash–treated soils can be effectively achieved through the formation of Pb silicate ($Pb_2SiO_4$). Palomo and Palacios[49] suggested that a different Pb silicate ($Pb_3SiO_5$) controls Pb immobilization in Pb-contaminated fly ash. Furthermore, Moulin et al.[50] reported that Pb immobilization was effective through the formation Si–O–Pb bonds. Further, Dermatas and Meng[12] showed that Pb was effectively immobilized by the formation of CSH compounds. Therefore, upon the addition of fly ash, Pb can be effectively immobilized by the formation of Pb silicate or pozzolanic reaction products, or both. Fly ash contains 23.2% CaO by weight; thus, the addition of fly ash provides lime to calcium-deficient sediment samples. The significant immobilization of metals may be attributed to the inherent alkalinity of fly ash.

## The Controlling Leaching Mechanisms

The controlling leaching mechanisms can be evaluated using the diffusion model developed by de Groot and van der Sloot[18]. The type of leaching mechanism that controls the release of metals can be determined based on the values of the slope of the logarithm of cumulative fraction release, log(*Bt*), vs. the logarithm of time, log(*t*), line[18], represented by Eq. 3. *Bt* is the cumulative maximum release of the component in mg $m^{-2}$ and *t* is the contact time in seconds.

When the slope is close to 1, according to the diffusion model[18], the process is defined as dissolution. In that case, the dissolution of the material from the surface proceeds faster than the diffusion through the pore space. If the slope is 0.5, the release of heavy metals will be slow and diffusion will be the controlling mechanism. Occasionally, a soluble layer exists on the surface of the material. During the initial phase of the leaching experiment, most of the soluble material in the soluble layer will be dissolved. This phenomenon is called surface wash-off and the process typically results in a slope close to 0. Typically, the long-term leaching characteristics of S/S-treated wastes are controlled by diffusion. However, there are cases when the other processes, dissolution and wash-off, may also occur[15]. It is important to determine the occurrence of dissolution and wash-off because they may lead to large amounts of the contaminant being released to the surrounding environment.

The controlling leaching mechanisms were evaluated using the described diffusion model[18] for the case of leaching with deionized water. The slope for all the sample plots and $R^2$ values are summarized in Table 2.

**TABLE 2**
**Regression Analysis Results for Cd and Pb Release**

| | **Cd** | | **Pb** | |
|---|---|---|---|---|
| | **Slope** | **$R^2$** | **Slope** | **$R^2$** |
| Untreated sediment | 0.18 | 0.98 | 0.23 | 0.96 |
| K10 | 0.15 | 0.99 | 0.44 | 0.98 |
| K20 | 0.18 | 0.99 | 0.61 | 0.93 |
| K30 | 0.53 | 0.76 | 0.35 | 0.92 |
| M10 | 0.53 | 0.99 | 0.35 | 0.96 |
| M20 | 0.65 | 0.99 | 0.36 | 0.83 |
| M30 | 0.65 | 0.99 | 0.35 | 0.94 |
| K10L10 | 0.43 | 0.99 | 0.46 | 0.96 |
| K20L10 | 0.41 | 0.99 | 0.43 | 0.96 |
| K30L10 | 0.65 | 0.99 | 0.35 | 0.96 |
| M10L10 | 0.56 | 0.99 | 0.35 | 0.95 |
| M20L10 | 0.62 | 0.99 | 0.45 | 0.97 |
| M30L10 | 0.56 | 0.99 | 0.41 | 0.91 |
| Z10 | 0.38 | 0.99 | 0.48 | 0.89 |
| Z20 | 0.35 | 0.99 | 0.50 | 0.85 |
| Z30 | 0.41 | 0.99 | 0.57 | 0.88 |
| F10 | 0.35 | 0.99 | 0.38 | 0.92 |
| F20 | 0.37 | 0.99 | 0.44 | 0.98 |
| F30 | 0.39 | 0.99 | 0.47 | 0.97 |
| Z5F10 | 0.60 | 0.99 | 0.51 | 0.92 |
| Z10F10 | 0.65 | 0.99 | 0.55 | 0.98 |
| Z5F20 | 0.60 | 0.99 | 0.54 | 0.99 |

For the untreated sediment sample, the slopes are 0.18 for Cd and 0.23 for Pb, indicating that surface wash-off was the main controlling mechanism of metal release. Moon and Dermatas[15] also reported this type of leaching mechanism for Pb from untreated soil samples.

Generally, the slope values obtained indicate that Pb and Cd released from treated samples were mainly controlled by diffusion. The slope values for most mixtures were in the range from 0.35 to 0.65 (Table 2), although there were exceptions for Cd in samples K10 (0.15) and K20 (0.18). Numerous researchers have previously arrived at the same conclusion. Côté et al.[51] showed that diffusion was the main controlling leaching mechanism for Pb release from a fly ash-lime–treated waste. Andrés et al.[52]

also demonstrated that diffusion was the dominant mechanism governing Pb release from stabilized steel foundry dusts.

## Cumulative Release of Cd and Pb Before and After S/S Treatment with AA and HA Solutions as Leachants

The percentages of Pb and Cd cumulatively leached with the AA solution pH 3.25 are higher than those obtained using deionized water, which is in concordance with the literature data related to the leachability of the other metals with solutions of lower pH[53]. The percentages of each metal extracted from an untreated sample were 22.3% for Cd and 15.2% for Pb, but for treated samples they were very low, ranging from 0.12 to 1.1% for Pb, and from 3.25 to 6.41% for Cd. This suggests that even at lower pH values, strong binding of Cd and Pb occurs by the solid constituents.

In this study, the ANS 16.1 method has been modified for the first time using HA solution as a leachant. Being anionic polyelectrolytes, humic substances can react with heavy metal ions, radionuclides, and many other environmental pollutants[54]. Even a low concentration of humic substances may significantly influence both free and total metal concentrations in sediments and groundwater[55], and thus play an important role in the binding and transport of metal ions in the environment. Therefore, the presence of humic substances can strongly influence the fate of metal ions, and potentially impact on the remediation of water or sediment polluted with heavy metals. If we compare the results of using HA as a leachant (mimicking landfill conditions), we can see that the percentages of Pb leached from all mixtures are very low (<1%), whereas those for Cd are somewhat higher (from 3 to 6%). If compared with deionized water, HA slightly increased water-soluble Cd. It seems that increased solubility of Cd is a consequence of its complexation with HA. Some other researchers have also reported that Cd is easily bound to organic matter, which increases its solubility, i.e., bioavailability[56]. On the other hand, HA restrains Pb availability[57]. In our case, it did not influence Pb solubility, which should be borne in mind when planning the remediation activities.

Generally, under the conditions mimicking real field conditions, the percentages of metals cumulatively leached from all mixtures were low.

## Effectiveness of S/S Treatments

Diffusion coefficients (De) for treated and untreated samples, computed by Eq. 1, are listed in Table 3. As can be seen from the table, the De values for treated samples were markedly lower than those for untreated samples. According to Nathwani and Phillips[22], diffusion coefficients generally range from $10^{-5}$ $cm^2$ $s^{-1}$ (very mobile) to $10^{-15}$ $cm^2$ $s^{-1}$ (immobile). The diffusion coefficients for Cd in treated samples ranged from $10^{-10}$ to $10^{-13}$ $cm^2$ $s^{-1}$ (low mobility) and for Pb from $10^{-13}$ $cm^2$ $s^{-1}$ (low mobility) to $10^{-15}$ $cm^2$ $s^{-1}$ (immobile). The Cd mobility was higher when leaching was carried out with the AA and HA solutions, whereas that for Pb was higher with the former leachant. This finding is in agreement with the results of cumulative release of the two metal ions from the S/S-treated mixtures.

The LX, which is a parameter directly derived from the ANS 16.1 test results, is currently used by Environment Canada[23] as a performance criterion for the utilization and disposal of treated waste. Treatment is considered effective if the LX value of treated waste is higher than 9 and S/S wastes are acceptable for a specific utilization, such as quarry rehabilitation, lagoon closure, roadbase material, etc. If the S/S wastes have an LX value higher than 8, they can be disposed of in segregated or sanitary landfills. If the S/S wastes have an LX value lower than 8, they are not considered appropriate for disposal and the immobilization treatment is considered ineffective. The effectiveness of S/S treatments using various agents was assessed by determining the LX values (Eq. 2). They were calculated using the diffusion coefficient obtained from Eq. 1 and are listed in Table 2. It is the average of the negative logarithm of the effective diffusivity terms (expressed in $cm^2$ $s^{-1}$).

**TABLE 3**
**Mean Effective Diffusion Coefficient (De) and LX**

| | Cd | | | | | | Pb | | | | | |
|---|---|---|---|---|---|---|---|---|---|---|---|---|
| | DI | | AA | | HA | | DI | | AA | | HA | |
| | Mean De ($cm^2$ $s^{-1}$) | Mean LX | Mean De ($cm^2$ $s^{-1}$) | Mean LX | Mean De ($cm^2$ $s^{-1}$) | Mean LX | Mean De ($cm^2$ $s^{-1}$) | Mean LX | Mean De ($cm^2$ $s^{-1}$) | Mean LX | Mean De ($cm^2$ $s^{-1}$) | Mean LX |
| Untreated sediment sample | 3.98E-7 | 6.4 | 7.90E-8 | 7.1 | 1.00E-7 | 7.0 | 2.50E-8 | 7.6 | 5.00E-8 | 7.3 | 3.90E-8 | 7.4 |
| K10 | 1.13E-11 | 10.95 | 3.16E-10 | 9.50 | 3.21E-10 | 9.49 | 1.22E-12 | 11.91 | 5.62E-12 | 11.25 | 1.09E-13 | 12.96 |
| K20 | 1.78E-11 | 10.75 | 3.19E-10 | 9.50 | 3.39E-10 | 9.47 | 1.18E-12 | 11.93 | 4.79E-12 | 11.32 | 7.91E-14 | 13.10 |
| K30 | 6.02E-13 | 12.22 | 2.95E-10 | 9.53 | 4.14E-10 | 9.38 | 3.53E-13 | 12.45 | 4.93E-13 | 12.31 | 4.24E-14 | 13.37 |
| M10 | 2.98E-11 | 10.53 | 3.03E-10 | 9.52 | 2.90E-10 | 9.54 | 3.58E-13 | 12.45 | 6.65E-13 | 12.18 | 7.24E-14 | 13.14 |
| M20 | 2.57E-11 | 10.59 | 3.45E-10 | 9.46 | 3.53E-10 | 9.45 | 1.15E-13 | 12.94 | 4.65E-13 | 12.33 | 3.88E-14 | 13.41 |
| M30 | 2.21E-11 | 10.66 | 3.80E-10 | 9.42 | 3.83E-10 | 9.42 | 1.04E-13 | 12.98 | 2.41E-13 | 12.62 | 2.85E-14 | 13.54 |
| K10L10 | 5.92E-11 | 10.23 | 2.12E-10 | 9.67 | 3.00E-10 | 9.52 | 8.93E-13 | 12.05 | 1.69E-12 | 11.77 | 9.90E-14 | 13.00 |
| K20L10 | 5.96E-11 | 10.22 | 1.89E-10 | 9.72 | 2.41E-10 | 9.62 | 8.70E-13 | 12.06 | 2.14E-12 | 11.67 | 1.39E-13 | 12.86 |
| K30L10 | 1.80E-10 | 9.74 | 7.49E-10 | 9.13 | 7.64E-10 | 9.12 | 3.13E-13 | 12.50 | 2.46E-12 | 11.61 | 1.52E-13 | 12.82 |
| M10L10 | 8.42E-11 | 10.07 | 2.77E-10 | 9.56 | 3.01E-10 | 9.52 | 4.56E-13 | 12.34 | 3.03E-12 | 11.52 | 3.67E-14 | 13.44 |
| M20L10 | 9.17E-11 | 10.04 | 2.19E-10 | 9.66 | 3.44E-10 | 9.46 | 8.98E-14 | 13.05 | 2.51E-13 | 12.60 | 1.11E-14 | 13.95 |
| M30L10 | 7.86E-11 | 10.10 | 2.50E-10 | 9.60 | 3.15E-10 | 9.50 | 1.72E-14 | 13.76 | 2.62E-13 | 12.58 | 3.99E-15 | 14.40 |
| Z10 | 3.82E-11 | 10.42 | 3.01E-10 | 9.52 | 3.63E-10 | 9.44 | 1.70E-13 | 12.77 | 3.91E-13 | 12.41 | 8.63E-14 | 13.06 |
| Z20 | 4.55E-11 | 10.34 | 4.46E-10 | 9.35 | 4.37E-10 | 9.36 | 1.80E-13 | 12.74 | 5.64E-13 | 12.25 | 7.98E-14 | 13.10 |
| Z30 | 2.64E-11 | 10.58 | 2.42E-10 | 9.62 | 2.63E-10 | 9.58 | 1.52E-13 | 12.82 | 2.89E-13 | 12.54 | 1.43E-13 | 12.85 |
| F10 | 4.65E-11 | 10.33 | 2.55E-10 | 9.59 | 3.06E-10 | 9.51 | 9.30E-14 | 13.03 | 4.81E-13 | 12.32 | 1.18E-13 | 12.93 |
| F20 | 5.37E-11 | 10.27 | 3.46E-10 | 9.46 | 3.76E-10 | 9.42 | 5.73E-14 | 13.24 | 6.29E-13 | 12.20 | 1.12E-13 | 12.95 |
| F30 | 7.12E-11 | 10.15 | 4.55E-10 | 9.34 | 4.31E-10 | 9.37 | 4.88E-13 | 12.31 | 8.25E-13 | 12.08 | 1.28E-13 | 12.89 |
| K5F10 | 8.85E-11 | 10.05 | 3.17E-10 | 9.50 | 3.31E-10 | 9.48 | 1.81E-14 | 13.74 | 1.09E-12 | 11.96 | 9.07E-14 | 13.04 |
| K5F20 | 1.02E-10 | 9.99 | 3.80E-10 | 9.42 | 3.28E-10 | 9.48 | 1.14E-13 | 12.94 | 1.05E-12 | 11.98 | 7.16E-14 | 13.15 |
| Z5F10 | 1.14E-10 | 9.94 | 3.91E-10 | 9.41 | 3.31E-10 | 9.48 | 9.66E-14 | 13.01 | 1.10E-12 | 11.96 | 5.05E-14 | 13.30 |
| Z10F10 | 1.05E-10 | 9.98 | 4.48E-10 | 9.35 | 3.51E-10 | 9.45 | 1.68E-14 | 13.77 | 6.31E-13 | 12.20 | 9.47E-14 | 13.02 |
| Z5F20 | 1.30E-10 | 9.89 | 4.11E-10 | 9.39 | 3.54E-10 | 9.45 | 1.56E-14 | 13.81 | 5.50E-13 | 12.26 | 4.88E-14 | 13.31 |

Since the LX values for treated samples are higher than 9, all of them can be considered acceptable for “controlled utilization”. Therefore, all the applied treatments of Pb- and Cd-contaminated sediments were effective in immobilizing metals. The LX values for Cd were lower, ranging from 9.74 (K30L10) to 12.22 (K30) compared to those for Pb (from 11.91, for K10 to 13.81 for Z5F20).

When the leachability test was performed with AA and HA solutions, the LX values were lower than those obtained using deionized water as leachant, ranging from 9.13 to 9.67 for Cd and from 11.25 to 14.40 for Pb. Since these values are greater than 9, on the basis of the adopted criterion, the treatments employed may be considered efficient, even under the conditions of sediment exposure to the influence of say acidic rains or increased organic loads.

A comparison of the concentrations obtained in the TCLP test[20] with the TCLP regulatory levels shows that the Pb and Cd concentrations are below maximum allowed values (Table 4). If these values are compared with the EPA limits[20], it comes out that all the tested element concentrations are within the EPA limits, which means that the heavy metals were successfully solidified into the S/S matrix. Also, the measured Cd and Pb concentrations were below the maximum allowed levels according to the Regulations for Surface Water[58]. Therefore, the treated sediment samples can be considered as nonhazardous, which shows the potential application of these S/S treatments.

Based on the LX values, all the applied treatments appeared to be efficient despite the fact that the two metals are differently distributed in the sediment. Still, if the results of sequential extraction of treated samples are compared with immobilization results, it can be seen that the diffusion coefficients obtained for Pb are smaller and LX values higher, so that it can be concluded that the treatments applied were more efficient for this metal. This observation can also be correlated with the results of sequential analysis since, based on them, in both original sediment and treated samples prior to leachability tests, the Pb was

**TABLE 4**
**Results of the TCLP Leaching Test (Mean Value ± RSD)**

| | Cd | Pb |
|---|---|---|
| | ($\mu$g l$^{-1}$) | |
| Untreated SS* | 1150 ± 35.2 | 5780 ± 245 |
| K10 | 104.8 ± 3.14 | 256.2 ± 4.25 |
| K20 | 114.7 ± 4.14 | 310.6 ± 2.23 |
| K30 | 91.18 ± 3.64 | 261.1 ± 4.12 |
| M10 | 79.95 ± 2.39 | 173.7 ± 2.98 |
| M20 | 35.72 ± 0.71 | 37.45 ± 0.44 |
| M30 | 78.39 ± 1.56 | 230.1 ± 4.56 |
| K10L10 | 69.81 ± 1.39 | 73.14 ± 1.59 |
| K20L10 | 86.45 ± 1.55 | 74.98 ± 1.68 |
| K30L10 | 123.4 ± 3.71 | 97.61 ± 0.99 |
| M10L10 | 57.02 ± 0.68 | 50.69 ± 1.02 |
| M20L10 | 135.2 ± 2.70 | 115.4 ± 3.21 |
| M30L10 | 82.94 ± 0.99 | 68.71 ± 2.51 |
| Z10 | 79.81 ± 1.59 | 171.9 ± 5.26 |
| Z20 | 67.49 ± 1.34 | 168.9 ± 4.31 |
| Z30 | 74.68 ± 1.11 | 205.1 ± 5.38 |
| F10 | 80.70 ± 1.12 | 180.1 ± 4.18 |
| F20 | 67.02 ± 1.30 | 195.6 ± 3.95 |
| F30 | 69.32 ± 0.98 | 191.2 ± 3.24 |
| Z5F10 | 112.1 ± 2.25 | 164.2 ± 2.25 |
| Z10F10 | 106.2 ± 1.27 | 201.2 ± 4.35 |
| Z5F20 | 101.3 ± 1.15 | 157.5 ± 2.47 |
| USEPA regulatory level ($\mu$g l$^{-1}$) | 1000 | 5000 |

* Untreated SS, untreated sediment sample.

already in the relatively inert fraction. As far as Cd is concerned, although the results of sequential analysis showed its presence in the exchangeable and reducible fractions, judging from the aspect of LX value and TCLP criterion, its immobilization was efficient.

## CONCLUSIONS

The assessment of the sediment quality based on the pseudo-total Pb and Cd content according to the corresponding Dutch standards and Canadian guidelines showed its severe contamination. The MW-assisted BCR sequential extraction procedure revealed different distribution of metals: Cd was dominant in the exchangeable and reducible fractions (37.7 and 60.87%, respectively) and Pb in the residual fraction (90.60%). The RAC reveals that Cd exists mostly in labile fractions and, therefore, is coming under the high-risk category and can easily enter the food chain. Because of its toxicity and availability, it can pose serious problems to the ecosystem. On the other hand, the proportion of Pb in the labile fraction in sediment is quite low and shows low risk to the aquatic environment.

All the S/S treatments applied appeared to be efficient in the remediation of sediment contaminated with Pb and Cd. The results showed that the efficiency of the applied remediation treatments may not be determined by different distribution of pollutants in sediment. Pb and Cd are completely differently distributed in sediments, but we applied the same remediation S/S treatments. Based on the LX values, diffusion coefficients, and the results of the TCLP test, applied S/S treatments are effective in immobilization of differently distributed metals, which is advantageous from an economic point of view, as this may justify the application of the already-expensive remediation procedures, especially when it comes to treat a material containing a mixture of pollutants.

In view of somewhat higher LX values, the treatments appeared to be more efficient in respect to Pb, which can be correlated to the results of sequential extraction of the starting sediment, since this, as well as treated samples, contains Pb in a relatively inert phase. Sequential extraction of treated samples prior to leachability tests showed a still-high percentage of Pb in the residual phase, whereas the Cd proportion even increased in the most mobile phase (up to 40%). The amount and type of clay appeared to be an important factor influencing Pb and Cd leachability. Montmorillonite samples showed a pronounced reduction in Pb and Cd leachability compared to kaolinite samples. This is most probably due to the larger surface area and the greater CEC of montmorillonite. The sole addition of fly ash, zeolites, and clays resulted in a significant decrease in the amount of Pb and Cd leached (reduced De and increased LX), compared to the untreated sample. The addition of these agents to the mixture caused a decrease in leachability except for the case of the mixture of zeolite and fly ash, whereby the increase in the fly ash proportion yielded a decrease of both leachability and diffusion coefficient, that is to the increase of LX value. Based on LX values, the most efficient mixture for Cd stabilization was M30 and for Pb Z5F20, whereas all treated samples were acceptable for “controlled utilization”. Since the LX values exceeded 9 even under the conditions mimicking those in sanitary landfill when such materials might be exposed to acidic rains or increased organic load, the controlling leaching mechanism was found to be wash-off. With the majority of samples, the controlling leaching mechanism of Pb and Cd upon the S/S treatment appeared to be diffusion. Therefore, only small amounts of metals would be expected to leach into the environment over time.

## ACKNOWLEDGMENT

This research was financed by the Ministry of Science and Technological Development of Republic of Serbia (Grants No. TR22012 and ON142058).
